# A Co-Association Network Analysis of the Genetic Determination of Pig Conformation, Growth and Fatness

**DOI:** 10.1371/journal.pone.0114862

**Published:** 2014-12-11

**Authors:** Anna Puig-Oliveras, Maria Ballester, Jordi Corominas, Manuel Revilla, Jordi Estellé, Ana I. Fernández, Yuliaxis Ramayo-Caldas, Josep M. Folch

**Affiliations:** 1 Departament de Ciència Animal i dels Aliments, Universitat Autònoma de Barcelona (UAB), 08193, Bellaterra, Spain; 2 Plant and Animal Genomics, Centre de Recerca en Agrigenòmica (CRAG), 08193, Bellaterra, Spain; 3 Génétique Animale et Biologie Intégrative UMR1313 (GABI), Institut National de la Recherche Agronomique (INRA), 78350, Jouy-en-Josas, France; 4 Génétique Animale et Biologie Intégrative UMR1313 (GABI), AgroParisTech, 78350, Jouy-en-Josas, France; 5 Laboratoire de Radiobiologie et Etude du Génome (LREG), Commissariat à l'énergie atomique et aux énergies alternatives (CEA), 78350, Jouy-en-Josas, France; 6 Departamento de Genética Animal, Instituto Nacional de Investigación y Tecnología Agraria y Alimentaria (INIA), 28040, Madrid, Spain; Wageningen UR Livestock Research, Netherlands

## Abstract

**Background:**

Several QTLs have been identified for major economically relevant traits in livestock, such as growth and meat quality, revealing the complex genetic architecture of these traits. The use of network approaches considering the interactions of multiple molecules and traits provides useful insights into the molecular underpinnings of complex traits. Here, a network based methodology, named Association Weight Matrix, was applied to study gene interactions and pathways affecting pig conformation, growth and fatness traits.

**Results:**

The co-association network analysis underpinned three transcription factors, *PPARγ*, *ELF1,* and *PRDM16* involved in mesoderm tissue differentiation. Fifty-four genes in the network belonged to growth-related ontologies and 46 of them were common with a similar study for growth in cattle supporting our results. The functional analysis uncovered the lipid metabolism and the corticotrophin and gonadotrophin release hormone pathways among the most important pathways influencing these traits. Our results suggest that the genes and pathways here identified are important determining either the total body weight of the animal and the fat content. For instance, a switch in the mesoderm tissue differentiation may determinate the age-related preferred pathways being in the puberty stage those related with the miogenic and osteogenic lineages; on the contrary, in the maturity stage cells may be more prone to the adipocyte fate. Hence, our results demonstrate that an integrative genomic co-association analysis is a powerful approach for identifying new connections and interactions among genes.

**Conclusions:**

This work provides insights about pathways and key regulators which may be important determining the animal growth, conformation and body proportions and fatness traits. Molecular information concerning genes and pathways here described may be crucial for the improvement of genetic breeding programs applied to pork meat production.

## Introduction

About 43% of the meat consumed worldwide proceeds from pigs, thus representing the major source of meat for human food intake [1]. Moreover, pig serves as a model for metabolic diseases such as obesity in humans [2], [3]. For meat industry, carcass conformation and growth are economically important traits, determining the proportions of the different commercial cuts [4]. Understanding the interactions between genes defining body growth and conformation of pigs is therefore critical for an efficient pig production.

Over 553 quantitative trait loci (QTLs) for growth-related traits have been reported in pigs [http://www.animalgenome.org/cgi-bin/QTLdb/SS/index]. Moreover, a genome wide linkage analysis for growth and body composition carried out in an Iberian × Landrace cross (IBMAP) confirmed previous QTL regions and identified new ones in 10 of the 18 autosomes [5]. Despite the large number of QTLs identified by QTL scan and Genome-Wide Association Studies (GWAS) the genetic architecture of these complex traits is far from being understood [6]. The detection of SNPs having a clear effect on complex traits using GWAS is limiting, still being a challenging task. The main reason is because many genes have a little effect, moreover, the need for multiple tests correction methods may result in removing some interesting SNPs [7]. The power of single trait GWAS can be enhanced when considering simultaneously multiple phenotypes because complex traits generally have multiple correlated traits [7].

Hence, for complex traits, a systems biology approach that integrates the results into coherent network models offers many advantages over single trait approaches [8]. Recently, a framework for integrating the information of GWAS with network inference algorithms, named Association Weight Matrix (AWM), was developed to reveal and identify key regulatory elements, provide *in silico* information and generate gene networks with the aim to better understand the regulatory mechanisms of complex traits [9], [10]. However, few studies have been performed to date using system biology approaches and genotypic data in livestock species [9], [11]–[15].

Network biology approaches may substantially improve our knowledge about the diverse molecular pathways underlying complex traits. Using this methodology, the main objective of this work was to identify key regulators, gene interactions and pathways determining pig growth and conformation traits in order to improve our knowledge about the architecture of these complex traits.

## Results and Discussion

### Global growth network description and trait cluster analysis

**Figure 1:**
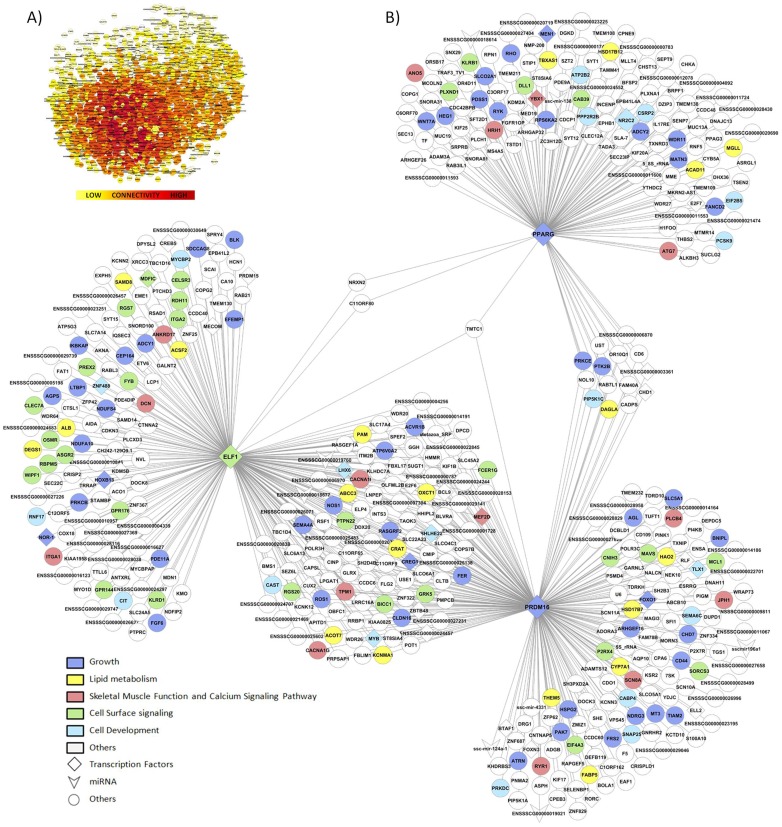
Co-association networks based on the AWM approach. **A**) Full network formed by 1,747 nodes, representing genes and SNPs, and a total of 316,166 edges, accounting for the interactions among them. **B**) Network formed by 513 nodes and 639 edges representing genes and interactions among the top trio of transcription factors. Colours corresponded to different functions according to the legend.

**Figure 2:**
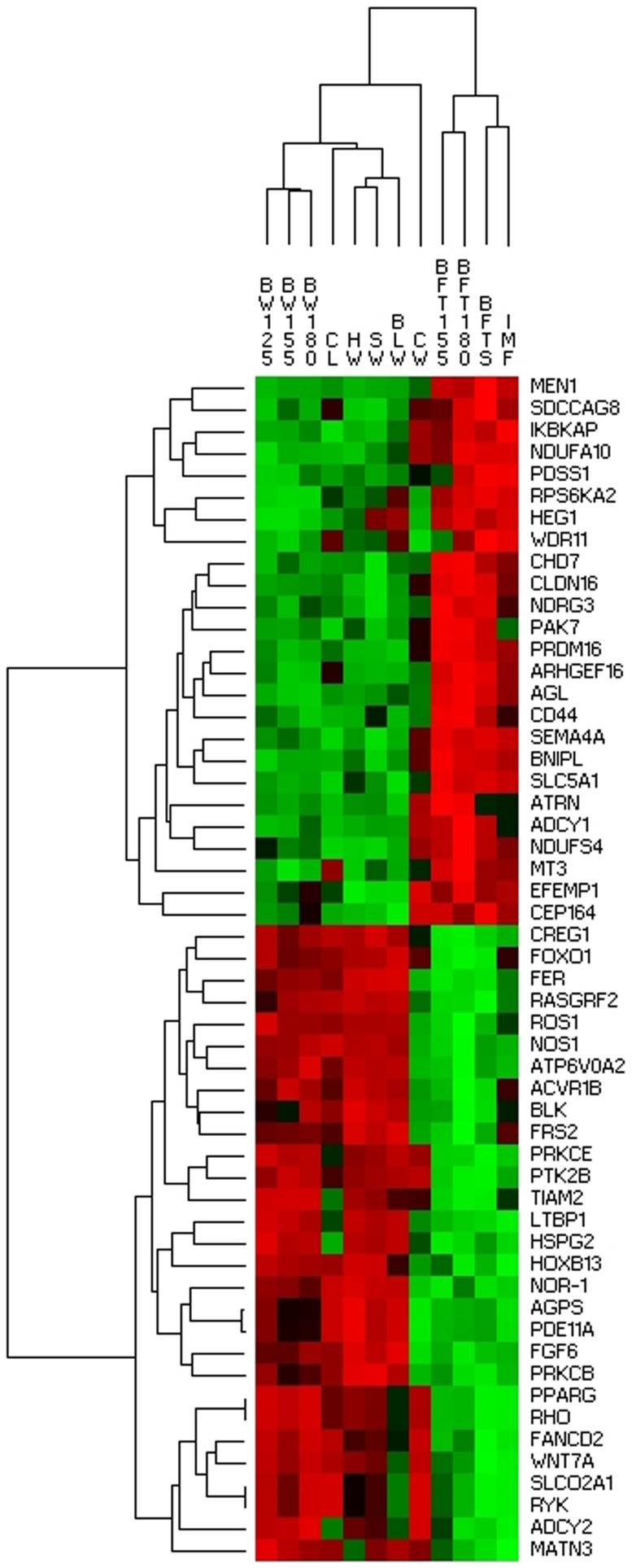
Hierarchical cluster analysis considering only those genes in the network related with growth (S1 Table) among 12 phenotypic traits. The green colour in the figure corresponds to negative SNP additive effect values and red to positive SNP additive effect values.

In the present study, we used a systems biology approach considering 12 growth-related phenotypes (Table 1). Given that primary cuts have economic impact in the Iberian pig production [16], ham weight was considered as the key trait for the AWM analysis. Among the genotypes of the 60K SNPs Porcine Beadchip, a total of 41,279 SNPs were retained for further analysis. Single-trait-single-SNP analysis by GWAS was performed for all traits (S1 Figure). The AWM approach captured a total of 1,747 annotated genes proximal to co-associated SNPs for conformation, growth and fatness traits. Therefore, an AWM with 1,747 nodes, representing genes, and a total of 316,166 edges, which account for the predicted interactions, was built (Fig. 1A). Interestingly, in the hierarchical cluster analysis two groups of phenotypic traits were formed showing a clear opposite directionality of the additive values. The first one containing the fatness traits (BFT155, BFT180, BFTS and IMF), whereas the second one encompassing the growth and conformation related traits (BW125, BW155, BW180, HW, SW, BLW, CW and CL) (S2 Figure). Additionally, a second cluster analysis considering only 54 genes (S1 Table) of the network which are known to be related with growth was performed, showing again, after clustering, two different groups for the additive values of growth and fatness traits (Fig. 2).

**Table 1:** Phenotypic traits registered in the BC1_LD (F1× Landrace) and in the BC (F2× Landrace) and F3 generations of the Iberian × Landrace cross.

Trait	Abbreviation	Statistics		
		N	Mean	SD
Body weight at 125 days (kg)	BW125	270	58.11	8.71
Body weight at 155 days (kg)	BW155	269	80.74	13.61
Body weight at 180 days (kg)	BW180	269	100.10	14.91
Carcass weight (kg)	CW	271	74.46	11.07
Carcass length (cm)	CL	261	81.86	6.23
Backfat thickness at 155 days (mm)	BFT155	269	13.34	3.08
Backfat thickness at 180 days (mm)	BFT180	220	15.60	3.21
Backfat thickness at slaughter (mm)	BFTS	237	23.26	6.13
Intramuscular fat percentage (%)	IMF	247	1.52	0.78
Weight of hams (kg)	HW	271	21.62	3.39
Weight of shoulders (kg)	SW	271	10.04	1.72
Weight of belly (kg)	BLW	276	7.33	1.14

Next, in order to simplify and visualize the data with Cytoscape software, the number of interactions was reduced by selecting only the strongest co-associations, major than 0.86 (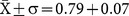). The resulting network had 53,200 predicted interactions and 1,703 genes.

### Key transcription factors regulating growth traits

Within the 1,703 associated-genes, a total of 142 putative regulators (S2 Table) were identified. After exploring all the possible interconnected trios among regulators, the top trio which spanned most of the network topology with highest connectivity (a total of 26,160 connections) and minimum redundancy was formed by the *Peroxisome Proliferator-Activated Receptor Gamma* (*PPARγ*; *PPARγ_Deg_*  = 147), the *E74-Like Factor 1* (*Ets Domain Transcription Factor*) (*ELF1*; *ELF1_Deg_*  = 237), and the *PR Domain Containing 16* (*PRDM16*; *PRDM16_Deg_*  = 256) genes. In the resulting network, there were a total of 639 co-associations with the top trio of TF connecting 513 genes (Fig. 1B). Interestingly, *ELF1* localized in a QTL on SSC11 identified for growth and body composition traits in the IBMAP cross [5], whereas no QTL was identified on SSC6 and SSC13 regions where the two other TF, *PRDM16* and *PPARG,* were located. This result supports that the network methodology allowed the detection of potential variations affecting the analyzed traits that would have not been detected by using single-trait based approaches (Table 2).

**Table 2:** Additive effect and p-value of the SNPs representing the top trio of transcription factors.

Gene	PRDM16		ELF1		PPARG	
Representative SNP	MARC0030882		MARC0000451		ISU10000701	
Trait	Additive Effect	P-value	Additive Effect	P-value	Additive Effect	P-value
BW125	−1.564	9.70E–02	1.767	2.89E–02	3.261	4.74E–05
BW155	−2.933	1.90E–02	2.217	4.09E–02	3.983	2.45E–04
BW180	−3.115	3.08E–02	1.762	1.59E–01	5.329	1.41E–05
CW	−0.734	5.23E–01	–0.008	9.95E–01	3.338	5.37E–04
CL	−0.534	1.28E–01	0.589	6.15E–02	0.758	1.26E–02
BFT155	0.630	4.78E–02	−0.011	1.00E+00	0.148	5.65E–01
BFT180	0.581	6.35E–02	−0.297	2.99E–01	0.161	5.54E–01
BFTS	0.666	3.38E–01	−0.526	3.46E–01	−0.537	3.73E–01
IMF	0.010	8.81E–01	−0.036	5.39E–01	−0.054	4.17E–01
HW	−0.580	1.16E–02	0.586	3.25E–03	0.546	6.01E–03
SW	−0.282	1.27E–02	0.229	2.19E–02	0.249	8.74E–03
BLW	−0.250	7.57E–03	0.174	3.24E–02	0.147	7.15E–02

In the network, *PPARγ* gene, which is a key regulator of adipocyte differentiation, glucose homeostasis and fatty acid metabolism, was highly connected presenting 147 co-associations with other genes. This gene plays a role in determining the energy balance and the fat deposition influencing growth and body size [17], [18]. Furthermore, *PPARγ* has been associated with obesity, diabetes and atherosclerosis [19], and it has been identified, using the same methodology, as a key transcription factor regulating cattle puberty-related traits [9]. Interestingly, in another study of our group, *PPARγ* was identified as over-expressed in pigs having more MUFA and SFA versus pigs with high PUFA content [20]. Other studies in pigs suggested that *PPAR*γ is an excellent target for determining growth and fat deposition traits at a certain age in pigs [21], [22].

On the other hand, *ELF1* gene is a major regulator of haematopoiesis and energy metabolism [23]. *ELF1* has also been described to trigger the *NF-κB* pathway activation involved in cell growth and differentiation and in lipid metabolism [24], [25]. Noteworthy, other members of the *ELF1* gene family (ETS transcription regulator family) are known to regulate adipocyte and osteoblast differentiation [26]. Remarkably, *FOXP3* which interacts with *ELF1* has been identified as a central transcription factor regulating IMF in cattle using the same methodology [13], [27].

Finally, *PRDM16* gene is involved in the differentiation of the brown adipose tissue, specifically in the switch between myogenic and adipogenic lineages [28]. *PRDM16* has been reported to control the myogenic cell fate into brown fat cells in mice, however, pigs lack in brown fat tissue [28], [29]. *PRDM16* can also function by triggering nervous and haematopoietic systems and participates in the regulation of the oxidative stress [30].

The embryonic mesoderm is a multipotent tissue that differentiates into myocytes, osteocytes and adipocytes [26]. The three top TF identified in the network have in common that are key regulators of the mesoderm cell fate. For instance, the over-expression of *PPARγ* may activate adipogenesis, *ELF1* may regulate adipocyte and osteoblast differentiation, meanwhile *PRDM16* may trigger the switch between adipose tissue and myocytes [28].

Selecting both miRNAs and TF as putative regulators did not affect the results, being the same top trio of genes identified as key regulators. In fact, inferring transcriptional and miRNA-mediated regulatory networks is still a challenge, particularly in non-model species such as the pig where the miRNA annotation is poor when compared to human or cow [31].

**Figure 3:**
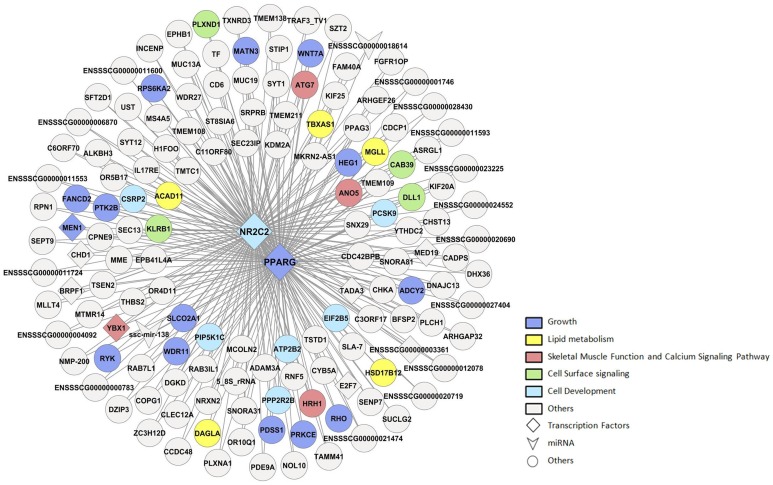
Network showing the shared-genes of *NR2C2* and *PPARγ*.

Additionally, a limitation of the AWM methodology is that only the nearest gene to the significant co-associated SNP is selected, discarding all other proximal genes. Linkage disequilibrium (LD) between molecular markers has to be taken into account for the AWM analysis. For instance, after exploring the network in more detail, a high co-association was observed between *Nuclear Receptor Subfamily 2, Group C, Member 2* (*NR2C2*) and *PPARγ* sharing the same co-associated nodes (Fig. 3). The strong relationship between *NR2C2* and *PPARγ* is supported by the literature, being *NR2C2* a repressor of *PPARγ* activity [32]. Interestingly, a deficiency of *NR2C2,* which has been suggested to play a critical role in the regulation of energy and lipid homeostasis, in mice causes growth retardation [32], [33]. Remarkably, *NR2C2* was also identified as co-associated in cattle growth network [12] and also in a network for fatness traits in cattle [13] and pig [11]. However, SNPs proximal to these genes (*PPARγ* and *NR2C2*) in our AWM network were separated by 1.27 Mb, being in complete LD (D′ = 1) (S3 Figure). Accordingly, LD can be a limitation to rule out which of these two genes play a key role regulating growth traits or if both genes are biologically relevant.

### Co-association network among the top TF

The parameters describing the network topology were calculated with CentiScaPe software, obtaining an average degree (Deg) of 62.44 and an average distance (AvD_G_) of 3.21; hence, showing a high degree of connection. A total of 54 genes out of the 513 nodes (Fig. 1B; blue colour in the network) belonged to growth-related gene ontologies (S1 Table). Noteworthy, a total of 20 genes were related to lipid metabolism (yellow colour in Fig. 1B). Among the 513 nodes setting up network connections, seven genes (*COPS7B, EFEMP1, ETV6, FRS2, HSPG2, SH3PXD2A* and *TGS1*) had been associated with human height, which is driven by growth and developmental processes [34], [35]. Interestingly, the *indian hedgehog* (*IHH*) gene product, identified in a human height GWAS study, binds to the patched domain containing 3 (PTCHD3) receptor, here identified as co-associated with *ELF1* [36]. In addition, 46 of the 513 genes were common with a study for cattle growth trait using the same methodology (*GRK5, NDRG3, RYK, FRS2, SCN8A, H1FOO, NALCN, EPB41L4A, LRRC16A, CNTNAP5, LTBP1, KHDRBS3, EPHB1, PRKCB, ATRN, TMEM108, PTK2B, RAPGEF5, RBPMS, SORCS3, SNX29, KCNN3, PLCH1, PLCB4, PDE11A, RGS7, NR2C2, WDR64, KCNMA1, DCN, SPEF2, CA10, MYB, RNF17, FYB, ETV6, CREB5, ZNF488, KSR2, SYT1, TBC1D16, SUCLG2, MLLT4, PLCXD3, VPS45, TUFT1*) [16]. Central to this network, *transmembrane and tetratricopeptide repeat containing 1* (*TMTC1*) gene appeared to be a common interaction factor for the 3 principal TFs. Not *TMTC1* but *transmembrane and tetratricopeptide repeat containing 2* (*TMTC2*) was also identified in the cattle growth network by Widmann *et al.* [12].

Aside from known interactions reported by the literature, our growth network allowed the identification of new interactions between genes that have not been previously described and may help in the understanding of such complex trait. In these sense, one of the top TF identified in the growth network, *ELF1* gene, has not been reported to date to be involved in growth processes. This gene was identified to be co-associated with *B Lymphoid Tyrosine Kinase* (*BLK*) gene in the AWM analysis. It has been reported that *ELF1* is a transcriptional activator of *BLK* and *SRC* kinases such as *v-yes-1 Yamaguchi sarcoma viral related oncogene homolog* (*LYN*) [37]. *BLK* is involved in the stimulation of insulin secretion in response to glucose [38]. In addition, the *SRC* family of protein tyrosine kinases (*SFKs*) interacts with growth factors [39] and cytokine receptors [40] and they are key mediators of PI3K and AKT signalling important for cell proliferation [41]. The *LYN* gene belonging to *SFKs* is required for rapid phosphorylation of Fer (Fps/Fes Related) Tyrosine Kinase (FER) [42]. In the AWM analysis we found *FER* also co-associated with *ELF1*.

Another interesting interaction identified in the co-association growth network was the *ELF1* with *HOXB13.* Supporting this interaction, it has been described that Myeloid ecotropic viral integration site 1 (MEIS1) is a HOX cofactor which is regulated by *ELF1* [43]. *HOXB13* may play a role in growth repression and spinal cord formation [44], [45]. Furthermore, *HOXB13* increases the androgen and favors the lipid accumulation in cells [46].

The transcription factor *Forkhead box O1* (*FOXO1*) gene, also identified in the network co-associated with *PRDM16*, is activated in response to glucocorticoids and is blocked via the IGF/Akt pathway. *FOXO1* is a target of insulin signalling and glucose metabolism, as well as it plays a role in myogenic growth and differentiation. Moreover, it has been observed that mice overexpressing *FOXO1* in skeletal muscle had a reduced skeletal muscle mass when compared with wild-type mice [47]. When FOXO1 and PPARGC1A act together they promote gluconeogenesis. *FOXO1* is known to repress *PPARγ* [48]. Another interesting gene was the miRNA ssc-miR-196a1, which was identified as co-associated with *PRDM16*, and has been recently reported to be associated with growth and development of skeletal muscle [49]. Other identified co-associated genes with *PRDM16* were *SH3PXD2A, ADAMTS12* and *PTPN22*. Interestingly, SH3PXD2A (SH3 And PX Domains 2A) is reported to bind the matrix metalloproteinases (ADAMs) and phosphoinositides [50]. Moreover, in human is associated with *ADAM12* (the membrane-anchored protein corresponding to the secreted protein ADAMTS12) which is involved in skeletal muscle regeneration and mediates the neurotoxic effect of beta-amyloid peptide [51]. Finally, *PTPN22* has been associated with diabetes in humans [52].

### Biological pathways and functional analysis

Functional analysis using IPA program allowed us to identify the biological functions overrepresented considering the 513 co-associated genes related to the top trio of TF. Among the networks identified with IPA there were: “cell signalling, nucleic acid metabolism, cell-to-cell signalling and interaction”, “organism development, DNA replication, recombination and repair, and lipid metabolism” and “hereditary disorder, neurological disease and developmental disorder”, all of them having a score  = 38, “cell-to-cell signalling and interaction, nervous system development and function, cellular assembly and organization” (score  = 33) and “cellular development, nervous system development and function, behaviour” (score  = 29) (S3 Table). Remarkably, the top molecular and cellular functions identified were: “post-translational modification” (p-value  = 9.01×10^−5^), “cell-to-cell signalling and interaction” (p-value  = 1.26×10^−4^), “molecular transport” (p-value  = 1.26×10^−4^), “cellular development” (p-value  = 2.89×10^−4^) and “cell morphology” (p-value  = 3.10×10^−4^). Among the top physiological system development functions we observed the “organismal survival” (p-value  = 1.18×10^−4^), “nervous system development and function” (p-value  = 1.26×10^−4^), “tissue development” (p-value  = 2.34×10^−4^) and “behaviour” (p-value  = 2.88×10^−4^). These results are in agreement with those obtained by Widmann *et al.* [12] using the same methodology to study growth traits in cattle, where they identified similar biological processes (cell communication, signal transduction, cellular process, cell surface receptor signalling pathway and cell adhesion) suggesting that different genetic variants may be affecting the same pathways even in different species.

Among the most overrepresented pathways identified we observed D-myo-inositol (1,4,5)-triphosphate (Ins(1,4,5)P_3_) biosynthesis (p-value  = 2.45×10^−4^), G-protein coupled receptor (GPCR) signalling (p-value  = 5.88×10^−3^), corticotropin releasing (CRH) hormone signalling (p-value  = 7.58×10^−3^), gonadotropin-releasing hormone (GnRH) signalling (p-value  = 1.55×10^−2^), caveolar-mediated endocytosis signalling (p-value  = 1.62×10^−2^), nuclear factor kappa-light-chain-enhancer of activated B cells (NF-κB) activation by viruses (p-value  = 1.62×10^−2^), phospholipase C (PLC) signalling (p-value  = 1.94×10^−2^) and neuronal nitric oxide synthase (nNOS) signalling in skeletal muscle cells (p-value  = 3.46×10^−2^) pathways (S4 Table). Noteworthy, in the cattle growth network study, the GnRH signalling and the nitric oxide (NO) pathway were also identified [16]. Some of these pathways (S4 Table) are discussed in more detailed below.

#### GPCR, PLC and Ins(1,4,5)P_3_ signalling pathways

In mammals during growth and development there is a high requirement of lipids to increase in cell size and number. Lipids are the primary substrates which bind to certain GPCRs leading to an induced activity of PLC, which catalyse the hydrolysis of phosphatidylinositol 4,5-bisphosphate (PIP_2_) to inositol 1,4,5-trisphosphate (IP_3_) and 1,2-diacylglycerol (DAG) both having important second messenger functions [53]. DAG can be also used as a component of biological membranes or as a precursor to triacylglycerol (TAG) for energy storage [54]. The IP_3_ molecule binds to the Ins(1,4,5)P_3_ receptors (InsP3R) and trigger Ca^2+^ channel opening activating the ryanodine receptor-operated channel (RYR) [55], [56]. The IP_3_ signalling mechanism is crucial for normal cell physiology [57]. Moreover, GPCRs jointly with phosphatidylinositol kinases (PIPK) may be involved in feed signal transduction pathways [58]. Two *PIPK* genes (*PIP5K1A* and *PIP5K1C*) jointly with *PLCB4* and *RYR1* were identified as co-associated to *PRDM16* gene. Thus, PRDM16 is a key transcriptional factor determining adiposity or miogenesis, and may also be necessary for the normal skeletal muscle development. Furthermore, the *GPCRs, GPR144* and *GPR176* were also identified in the growth network.

#### GnRH and CRH signalling pathway

Two hormone-related pathways, GnRH and CRH signalling pathways, were identified as overrepresented in our data. CRH is a peptide hormone secreted by the hypothalamus which controls adrenal secretion of cortisol and has been suggested to play a role in cell growth and survival. Interestingly, sheep with high cortisol response were prone to obesity [59]. Moreover, it has been observed that children treated with glucocorticoids showed growth retardation [60]. GnRH, which binds to GNRHR (GPCR member 7), is synthesized and released from neurons within the hypothalamus and regulates the production of gonadotropins, such as luteinizing hormone (LH) and follicle-stimulating hormone (FSH) in the pituitary gland, which, in turn triggers sexual maturation and promotes the secretion of endogenous sex hormones such as testosterone and estrogen from the gonads. Interestingly, growth-related traits in pig depend largely on gender [61]. In fact, GnRH agonists are used to treat central precocious puberty (CPP) characterized for developing an early puberty, larger growth of the skeleton and adult height [62]. Besides, the GnRF analog-diphtheria toxoid conjugate is used for castration and it is known to increase body weight at slaughter and improve average daily gain and feed conversion ratio [63]. The release of Ca^2+^ and DAG enhances the activation of protein kinases (PKC) to increase gonadotropin hormone secretion. Interestingly, in the network we can observe *PRKCB* co-associated to *ELF-1* and *PRKCE* co-associated with *PPARγ* and *PRDM16*. These results underline an important function of the three key TF in our network having an important role in the puberty and bone growth development.

#### NF-κB and NO signalling pathway

NF-κB is a pleiotropic transcription factor being involved in many biological processes such as inflammation, immunity, differentiation, cell growth and apoptosis. This signalling pathway has also been identified in a study of human growth using gene expression data [64]. It is reported that *ELF1* interacts with *NF-κB* via DNA binding domain [37]. Furthermore, the NF-κB activity may activate nNOS to generate NO [65]. NO may trigger GH secretion and affects other several pituitary peptides such as gonadotropins [66]. It is reported that a chronic exposure of NO may stimulate angiogenesis and adipocyte development [67]. Interestingly, we observed *NOS1* co-associated with *ELF1* in the growth network (Fig. 1B). Both, *ELF1* and *NOS1* genes may play a role in hematopoiesis and vascular development [68]. On the other hand, NO has the ability to enhance and regenerate diseased muscle, through determining the fibro-adipogenic progenitors fate and inhibiting adipogenesis [69]. In this direction, the NO production is thought to inhibit *PPARγ* expression [69]. Surprisingly, *NOS1* was also identified as co-associated with *PRDM16*, but not with *PPARγ*.

### General discussion: from network to phenotype inference

Iberian pigs are known to have higher IMF than Landrace pigs at the same growth stage [70]. Furthermore, the skeletal muscle grows faster in Landrace than in Iberian pigs, being less prone to obesity [70]. In this study, we analyzed backcrossed animals from an Iberian × Landrace cross, which differed in fat and growth traits. Growth refers to an increase in tissue mass and it can be plotted as a sigmoid curve depending on age and cumulative weight [71]. At the pre-mature phase, muscle mass, organ and bone formation are increased, meanwhile in the mature phase the animal is more prone to fattening and intramuscular deposition [71]. The top trio of TF identified in the network are key regulators for mesoderm cell differentiation in osteocytes, miocytes or adipocytes. Our results showed among the top molecular and cellular functions the cell development and interaction pathways which may be important in order to trigger tissue formation. Noteworthy, the hierarchical cluster analysis evidenced a clear division of the additive effects of the SNPs for the 12 growth phenotypic traits, between animal weight-related and fat-related traits (S2 Figure and Fig. 2). We hypothesize that the hormone releasing pathways here identified (GnRH and CRH) may be key for the regulation of conformation, growth and fatness traits in our animal material. An increased carcass weight with a reduced backfat thickness at a fixed age have been selection targets in commercial pig breeds, resulting in less mature animals as fat deposition rate is expected to increase in the puberty phase [72]. Given that Iberian breed is more prone to IMF and backfat deposition at the same growth stage lead to the hypothesis that Landrace animals may arrive later to the mature growth phase when compared to Iberian animals. What remains unclear is whether the signals for maturity switching are related to the GnRH or CRH hormone release pathways. Finally, the genes and pathways here identified had a high concordance with those reported by other authors studying growth metabolism in animals or height related traits in human. Our hypothesis are supported by the high concordance between our genes and pathways identified in the network and those reported by Fortes *et al.* [9] for puberty traits in cattle.

### Conclusions

The processes regulating conformation, growth and fatness traits in pigs are complex and most of the mechanisms remain unknown despite being of great interest for the pig industry. The power of single trait GWAS can be enhanced when considering simultaneously multiple phenotypes taking advantage of system biology approaches. In the present study, the AWM gene co-association network analysis revealed key transcription factors, gene-gene interactions and pathways underpinning the regulation of pig conformation, growth and fatness. Network approaches represent a major step in understanding the genetics of complex diseases and traits. Further efforts should be made in order to study in more detail the new gene-gene interactions here identified, as well as, to study in more detail the key transcription factors and pathways involvement in the growth and conformation traits determination.

## Material and Methods

### Animal material and phenotypic classification

The animal material used belongs to several generations of the IBMAP population obtained from the cross of 3 Iberian boars (Guadyerbas) with 31 Landrace sows [73], [74]. For this study we used phenotypic records from 292 animals belonging to three different IBMAP generations: 159 BC1_LD animals (25% Iberian ×75% Landrace) from backcrossing five F1 males with 26 Landrace sows, 79 BC animals obtained by crossing 4 F2 boars and 22 Landrace sows and 54 F3 obtained by mating F2 animals. Animals were fed *ad libitum* and sacrificed at 180±2.8 days (average ± standard deviation) in a commercial slaughterhouse following national and institutional guidelines for the Good Experimental Practices and approved by the Ethical Committee of the Institution (IRTA- Institut de Recerca i Tecnologia Agroalimentàries).

Phenotypic records used in the analyses (Table 1) correspond to body weight (BW) measured at 125, 155 and 180 days (BW125, BW155, and BW180, respectively), backfat thickness (BFT) at the level of the fourth rib at 4 cm of the midline measured by ultrasounds at 155 and 180 days (BFT155 and BFT180) and measured with a rule at slaughter (BFTS), carcass length (CL) and carcass weight (CW), ham weight (HW), shoulder weight (SW), belly weight (BLW), and the intramuscular fat content (IMF) in the *longissimus dorsi* muscle.

### Genetic markers and quality control

A total of 364 pigs, including their F0, F1 and F2 founder generations (72 animals), were genotyped with the Porcine SNP60K BeadChip [75] following the Infinium HD Assay Ultra protocol (Illumina Inc.; San Diego, CA, USA) and the genotypes were visualized with the GenomeStudio software (Illumina Inc.; San Diego, CA, USA). The quality control of the 62,163 SNPs was performed by using Plink [76] software removing markers with a minor allele frequency (MAF) <5% and animals with missing genotypes>5%. The SNP mapping and annotation was performed by using the pig assembly 10.2 [ftp://ftp.ncbi.hlm.nih.gov/genomes/Sus_scrofa/GEF/]. We also excluded markers which did not map in the Sscrofa10.2 version assembly. Pedstats program [77] was used to check Mendelian inheritance errors.

### Genome-wide association analysis

Genome-wide association analysis (GWAS) for the twelve phenotypic growth traits were performed using a mixed model accounting for additive effects with Qxpak 5.0 software [78]:



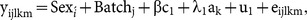
in which y_ijlkm_ was the i-th individual record, sex (two levels) and batch (nine levels) were fixed effects, ß was a covariate coefficient with *c* being the covariate used in each case (described below), λ_l_ was a −1, 0, +1 indicator variable depending on the l-th individual genotype for the k-th SNP, a_k_ represented the additive effect associated with the k-th SNP, u_l_ represented the infinitesimal genetic effect with random distribution N(0, 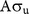) where **A** was a numerator of the the pedigree-based relationship matrix and e_ijlkm_ the residual.

Different covariates (*c*) were used for the analysis. Carcass weight was used as a covariate for CL, IMF, BFTS, HW, SW, and BLW. For BFT155 and BFT180 the covariates used were the body weight at their respective days. Meanwhile, for the body and the carcass weights the covariate used was the animal age.

### Association weight matrix

The association weight matrix (AWM) was built from the GWAS results. First, the SNP additive effects were normalized with a z-score method using a R script and a matrix was constructed with these values, being SNPs in rows and traits in columns. Another matrix with the same format was generated for the p-values obtained in the GWAS. For the analysis, the ham weight was selected as the key phenotype. Subsequently, the AWM script [9] available from authors was used in R (http://www.r-project.org/). Those SNPs associated (nominal p-value <0.05) with the ham weight or with 3 or more traits were selected for further analysis. We included in the analysis the SNPs with a distance of minor than 2.5 kb (SNPs close) and major than 1,000 kb (SNPs far) from a gene. We also included SNPs located at less than 10 kb of miRNA. Finally, to facilitate the analysis, for SNPs clustering at less than 1 Mb of distance from each other, the SNP associated with the major number of characters was selected. The hierarchical clustering option of PermutMatrix software [79] was used to visualize the results of both traits and genes. The trio of putative regulators spanning most of the network topology with a minimum redundancy [10] was selected. In this study we took into account all the transcription factors (TF) from the list reported by Vaquerizas *et al.* [80]; additionally, those 22 genes belonging to the GO: 0050789 which accounts for the DNA binding TF activity were added. All miRNA annotated on Sscrofa10.2 assembly were also included in the analysis as potential regulators. PCIT algorithm [81] was used to construct a file containing the reported gene-gene interactions among the 3 TFs. The CentiScaPe plug-in [82] of Cytoscape software [83] was used to visualize the PCIT results and either to calculate the node centrality values (Deg) and network parameters.

### Gene ontologies, pathways and network analysis

The Ingenuity Pathways Analysis software (IPA; Ingenuity Systems, Redwood city, CA, USA; www.ingenuity.com) was used to identify the most relevant biological functions and pathways in which the genes associated with the phenotypic traits were involved. IPA, which uses its own databases, allowed the identification of overrepresented pathways using the BH multiple testing correction [84] of p-value (FDR <0.05) and generating biological networks. The Mouse Genome Database (MGD; http://www.informatics.jax.org) was used in order to identify how mutant alleles driven in mice for the identified growth-related genes present in the network affected the phenotype.

## Supporting Information

S1 Figure **GWAS plot of the 12 traits: body weight measured at 125, 155 and 180 days (BW125, BW155, and BW180, respectively), backfat thickness measured at 155 and 180 days (BFT155 and BFT180) and measured at slaughter (BFTS), carcass length and weight (CL and CW), weight of the hams, shoulders and belly (HW, SW and BLW) and intramuscular fat (IMF) content.** The horizontal green line represents the statistical significance (false discovery rate; set at q-value ≤0.05) calculated with the q-value library [85] implemented in R program (http://www.r-project.org/).
(DOCX)

S2 Figure **Hierarchical cluster analysis among 12 phenotypic traits: body weight measured at 125, 155 and 180 days (BW125, BW155, and BW180, respectively), backfat thickness measured at 155 and 180 days (BFT155 and BFT180) and measured at slaughter (BFTS), carcass length and weight (CL and CW), weight of the hams, shoulders and belly (HW, SW and BLW) and the intramuscular fat (IMF) content.**
(TIF)

S3 Figure **Linkage disequilibrium among the** ***PPARG*** **and** ***NR2C2*** **SNPs. Pattern of linkage disequilibrium analysis around ±2Mb of the SNPs in** ***PPARG*** **and** ***NR2C2*** **.** Figure colored from blue to red according to LD strength between consecutive markers. The green diamond-shape corresponds to the SNP in *PPARG* gene and the blue diamond-shape the SNP in *NR2C2* gene.
(DOCX)

S1 Table **List of 54 growth-related genes in the network.**
(XLSX)

S2 Table **List of 142 regulators (transcription factors and miRNAs) identified within the list of associated-genes.**
(XLSX)

S3 Table **Top networks of molecular functions identified with IPA for the 513 genes.**
(XLS)

S4 Table **Top pathways identified with IPA for the 513 genes.**
(XLS)
