# Supplementary material for: A Co-Association Network Analysis of the Genetic Determination of Pig Conformation, Growth and Fatness
Source: PLoS One. 2014 Dec 11;9(12):e114862. doi: 10.1371/journal.pone.0114862 (PMC4263716; doi:10.1371/journal.pone.0114862)

**Figure S1.** GWAS plot of the 12 traits: body weight measured at 125, 155 and 180 days (BW125, BW155, and BW180, respectively), backfat thickness measured at 155 and 180 days (BFT155 and BFT180) and measured at slaughter (BFTS), carcass length and weight (CL and CW), weight of the hams, shoulders and belly (HW, SW and BLW) and intramuscular fat (IMF) content. The horizontal green line represents the statistical significance (false discovery rate; set at q-value ≤ 0.05) calculated with the q-value library [85] implemented in R program (http://www.r-project.org/).

1. BW125


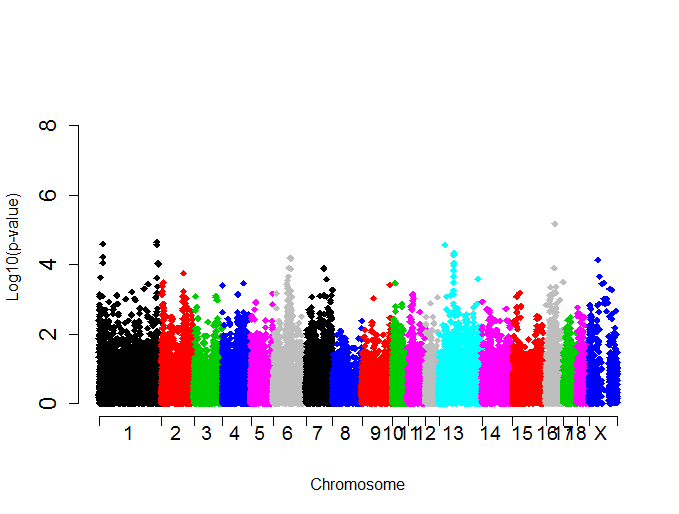


1. BW155


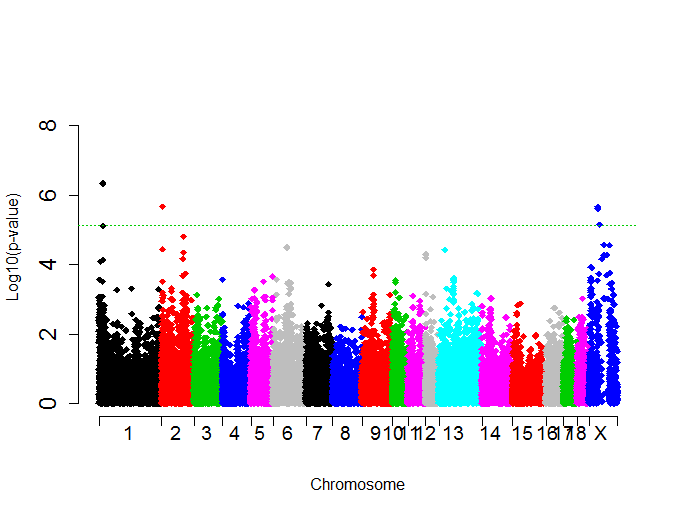


1. BW180


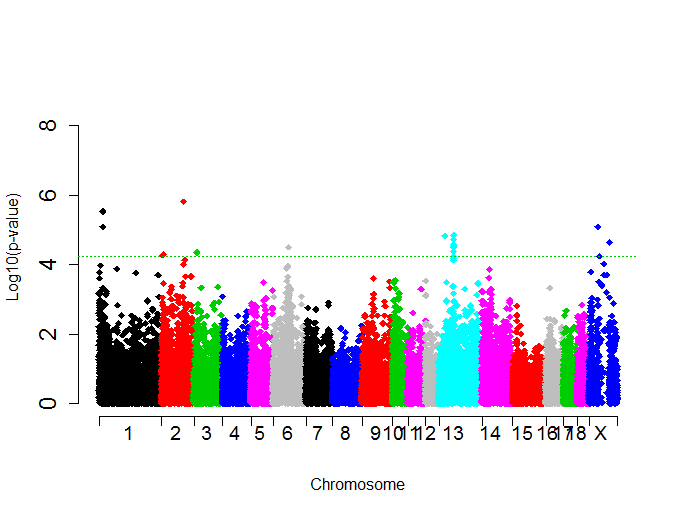


1. CW


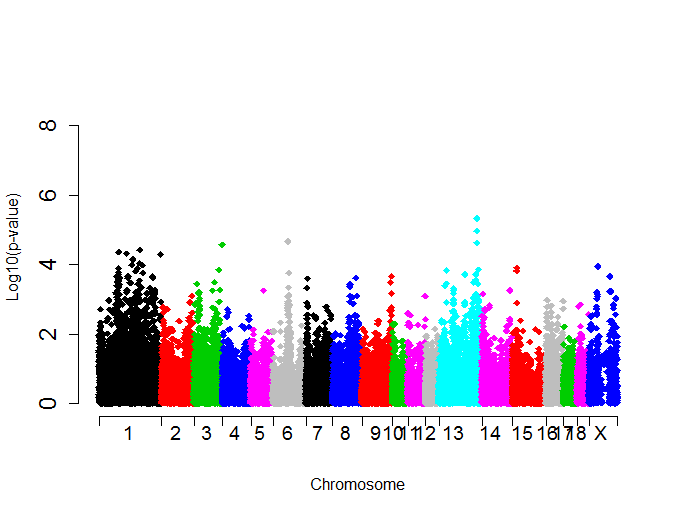


1. CL


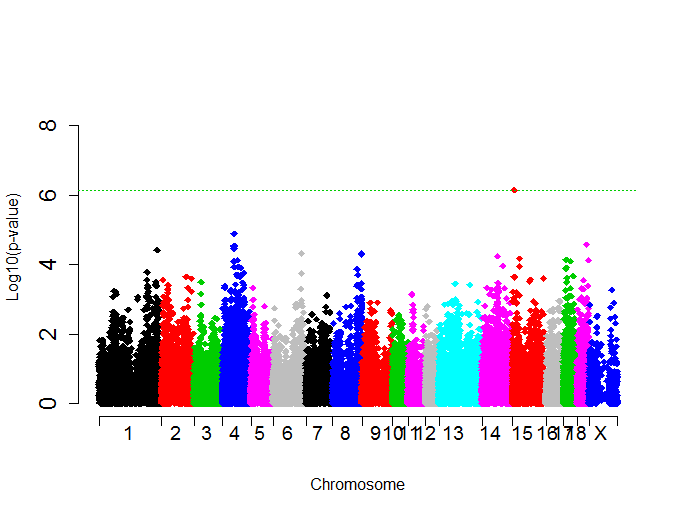


1. BFT155


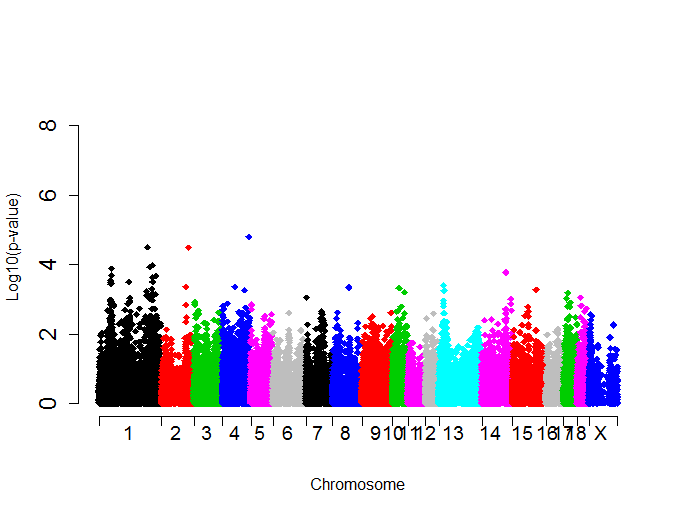


1. BFT180


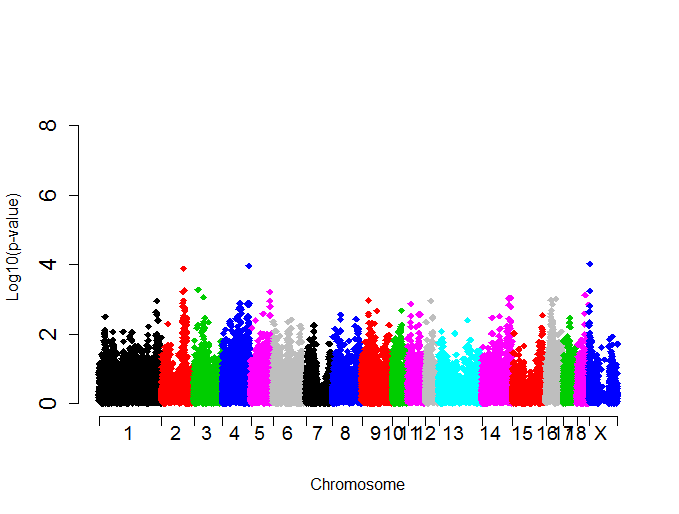


1. BFTS


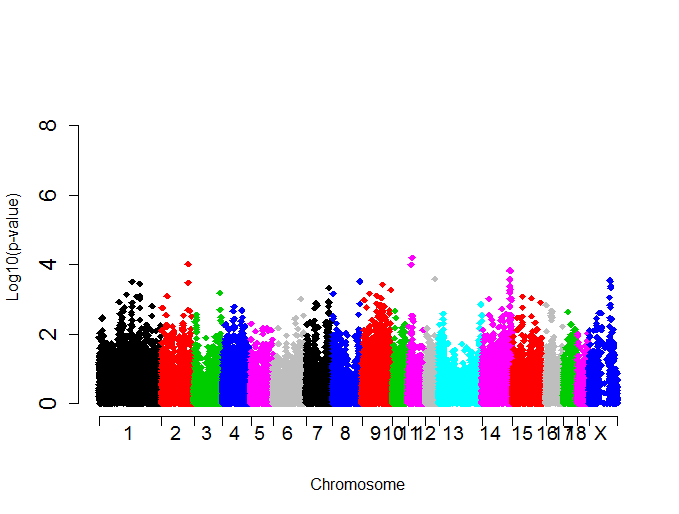


1. IMF


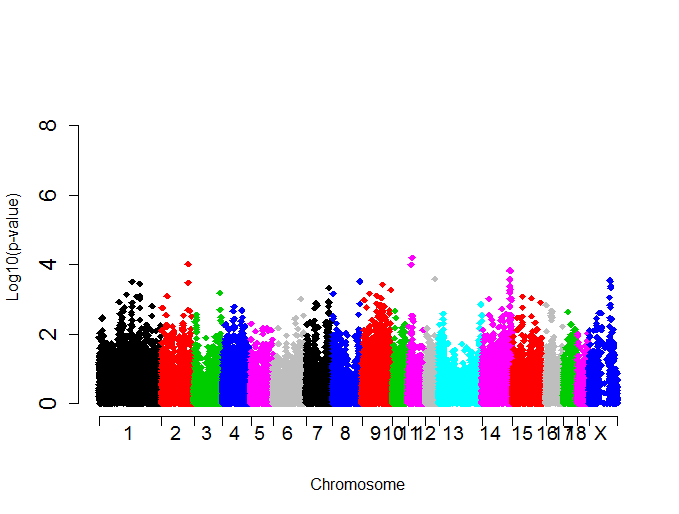


1. HW


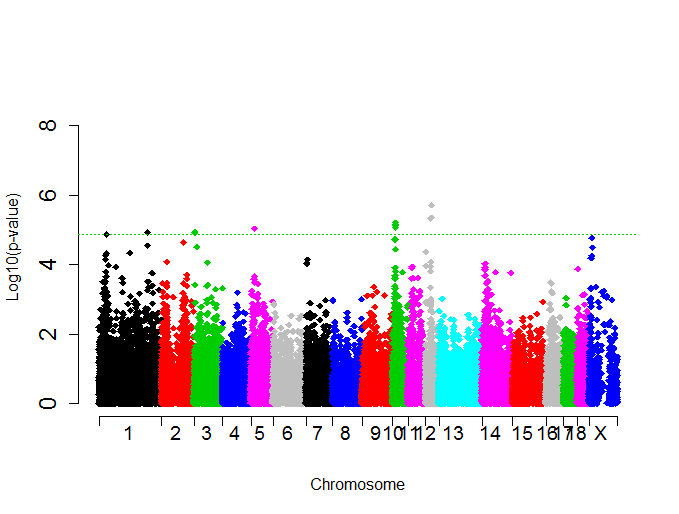


1. SW


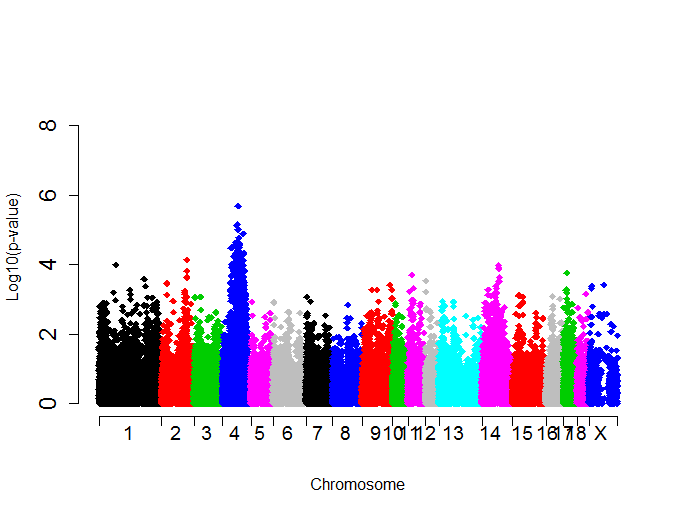


1. BLW


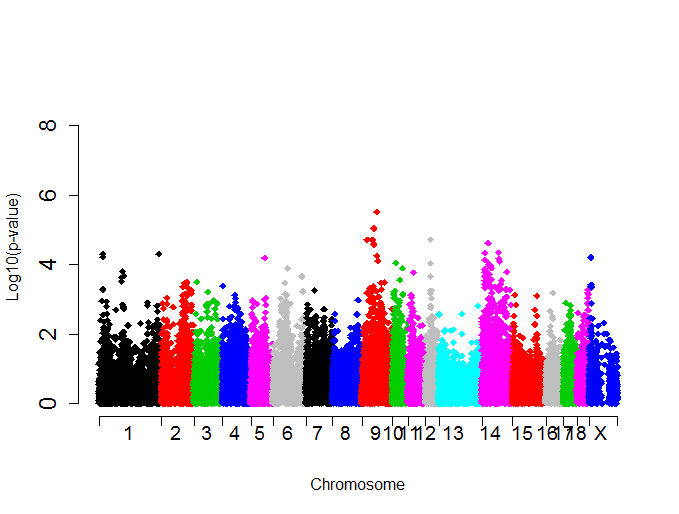

Supplement: S1 Figure — GWAS plot of the 12 traits: body weight measured at 125, 155 and 180 days (BW125, BW155, and BW180, respectively), backfat thickness measured at 155 and 180 days (BFT155 and BFT180) and measured at slaughter (BFTS), carcass length and weight (CL and CW), weight of the hams, shoulders and belly (HW, SW and BLW) and intramuscular fat (IMF) content. The horizontal green line represents the statistical significance (false discovery rate; set at q-value ≤0.05) calculated with the q-value library [85] implemented in R program (http://www.r-project.org/). (DOCX) [file pone.0114862.s001.docx]
