# Supplementary material for: A Co-Association Network Analysis of the Genetic Determination of Pig Conformation, Growth and Fatness
Source: PLoS One. 2014 Dec 11;9(12):e114862. doi: 10.1371/journal.pone.0114862 (PMC4263716; doi:10.1371/journal.pone.0114862)
Supplement: S3 Figure — Linkage disequilibrium among the PPARG and NR2C2 SNPs. Pattern of linkage disequilibrium analysis around ±2Mb of the SNPs in PPARG and NR2C2 . Figure colored from blue to red according to LD strength between consecutive markers. The green diamond-shape corresponds to the SNP in PPARG gene and the blue diamond-shape the SNP in NR2C2 gene. (DOCX) [file pone.0114862.s003.docx]

**Figure S3.** Linkage disequilibrium among the *PPARG* and *NR2C2* SNPs. Pattern of linkage disequilibrium analysis around ±2Mb of the SNPs in *PPARG* and *NR2C2*. Figure colored from blue to red according to LD strength between consecutive markers. The green diamond-shape corresponds to the SNP in *PPARG* gene and the blue diamond-shape the SNP in *NR2C2* gene.

**
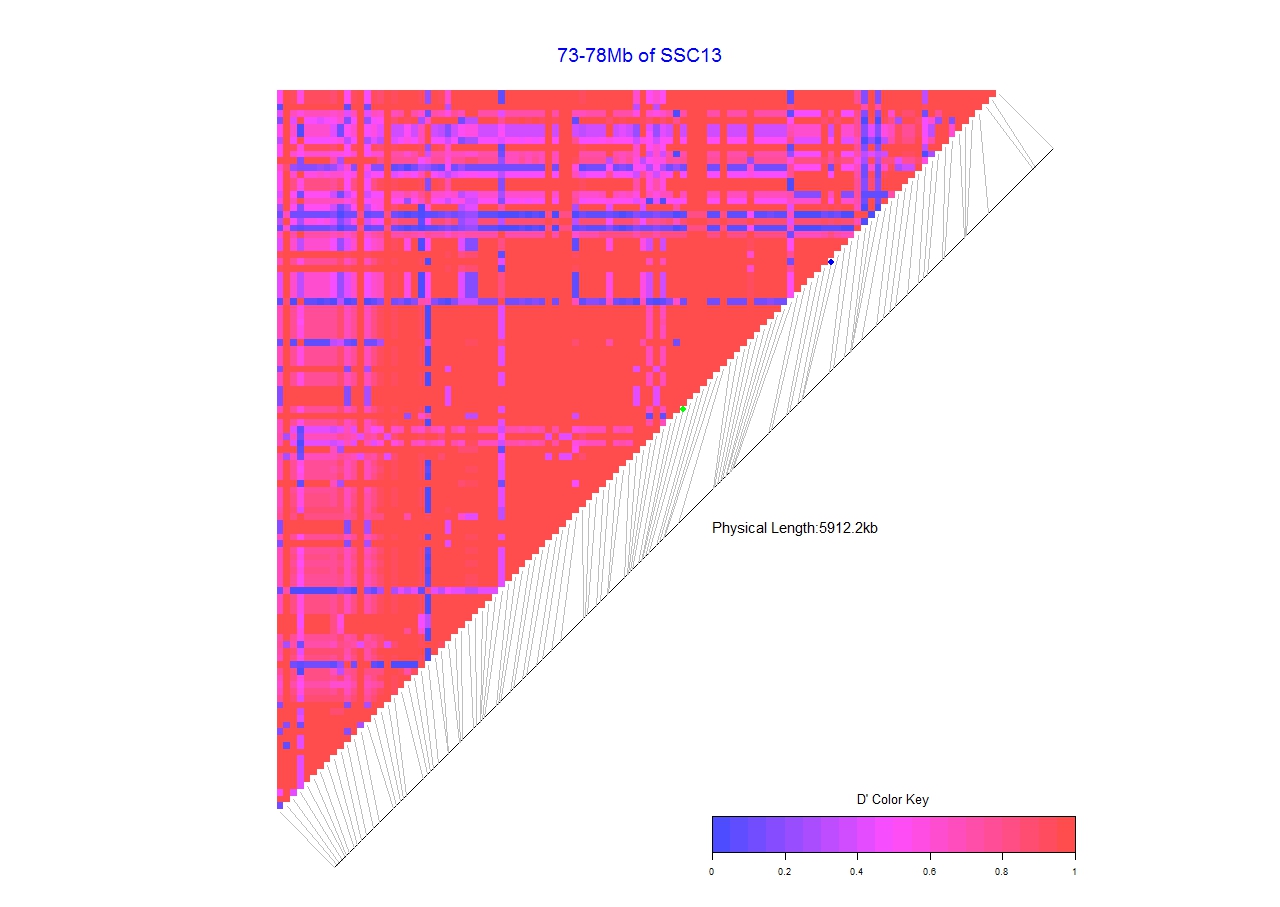
**
